# ATP/P2X7 receptor signal aggravates ischemic stroke injury by activating Th17 cells via STAT3/IL-21 pathway

**DOI:** 10.3389/fimmu.2025.1558307

**Published:** 2025-08-28

**Authors:** Wenying Liu, Denghui Li, Mengjie Zhang, Jun Yin, Peng Wang, Paiyu Liu, Zhiqiang Song, Bing Ni, Yanmeng Peng

**Affiliations:** ^1^ Department of Pathophysiology, College of High Altitude Military Medicine, Army Medical University (Third Military Medical University), Chongqing, China; ^2^ Department of Dermatology, Southwest Hospital, Army Medical University (Third Military Medical University), Chongqing, China; ^3^ Department of Rehabilitation, Southwest Hospital, Army Medical University (Third Military Medical University), Chongqing, China

**Keywords:** ATP, P2X7 receptor, ischemia stroke, CD4 + T cells, IL-17A, IL-21, P-STAT3

## Abstract

**Background:**

During cerebral ischemia, adenosine triphosphate (ATP) is released into the extracellular matrix from damaged neurons and glial cells, functioning as a danger signal. However, the involvement of ATP/P2X7 signaling in regulating the infiltrated lymphocytes during ischemia-reperfusion (IR) injury remain unclear.

**Methods:**

The expression level of P2X7 was evaluated in infiltrated lymphocytes from experimental stroke mice. To further elucidate the role of P2X7 signaling in infiltrated immune cells during ischemic stroke, P2X7-knockout (KO) mice and Rag2^-/-^ mice were utilized. Additionally, *in vitro* experiments were conducted to explore the underlying mechanisms.

**Results:**

Flow cytometry analysis revealed that the expression of P2X7 was mainly expressed in CD4^+^and CD8^+^T cells among the infiltrated lymphocytes in stroke lesions of the mice. P2X7-KO mice exhibited smaller infarct sizes and improved neurological function compared to wild-type mice. Rag2^-/-^ mice that received P2X7-KO CD4^+^T cells demonstrated reduced ischemic-reperfusion injury and a decreased level of IL-17A and frequency of Th17 cells compared to Rag2^-/-^ mice that received wild type CD4^+^T cells. Transcriptome sequencing and *in vitro* experiments indicate that P2X7 may mediate the expression of IL-21 and regulating the synthesis of IL-17A and the differentiation of Th17 cells. We also confirmed that P2X7 receptor regulates IL-21 through STAT3 signaling.

**Conclusions:**

Our findings suggest that the loss of ATP/P2X7 signaling in CD4^+^T cells may inhibit the pSTAT3, IL-21 pathway, leading to reduced differentiation of Th17 cells and ultimately mitigating IR injury. This provides novel insights into the role of ATP/P2X7-mediated signaling in T cell inflammation during ischemic stroke.

## 1 Introduction

Neurons with high demands for glucose and oxygen are particularly susceptible to ischemic injury. Disruption of energy metabolism during ischemic stroke results in ionic imbalance, mitochondrial dysfunction, oxidative stress-induced damage, and ultimately leads to cellular excitotoxicity and cell death (1). Neuronal cell death triggers the release of damage-associated molecular patterns (DAMPs), which can induce localized inflammatory responses (2). Among these DAMPs, ATP is notably released into the extracellular matrix during cerebral ischemia, leading to a significant increase in extracellular ATP (eATP) concentrations at the site of stroke lesions (3, 4). The P2X7 receptor (P2X7 or P2X7R) is an ATP-gated non-selective cation channel. Activation by ATP leads to the opening of the P2X7 channel pore, facilitating the influx of Ca^2+^ and Na^+^ as well as the efflux of K^+^, thereby initiating downstream signaling cascades (5).

P2X7 has low ATP affinity and is predominantly activated by high extracellular ATP concentrations typically under pathophysiological conditions. At low ATP concentrations, P2X7 receptors are mildly activated, functioning as small cation channels (6). Continuous or repeated exposure to high level of ATP causes a large, irreversible, non-selective pore, triggering downstream effects like altered membrane permeability, cell death, inflammation, metabolic changes, and cell fate decisions (7). P2X7 receptors are expressed in glial and neuronal cells (8). Following ischemic stroke, the expression of the P2X7 receptor is upregulated (9). Enhanced P2X7 signaling mediated activation of CNS cells and involved in both the injury and tolerance of ischemic stroke (9).

Cerebral ischemia disrupts the blood-brain barrier, permitting circulating immune cells to infiltrate ischemic brain tissue. During the acute and subacute phases post ischemia stroke, infiltrated lymphocytes aggravating the neurological damage. CD4^+^T cells including Th1 and Th17 cells contribute to secondary injury by releasing pro-inflammatory cytokines, damaging neurons, activating glial cells, breaking down the blood-brain barrier and bringing in more peripheral immune cells (10, 11). CD8^+^T cells can cause neuron necrosis through cell interactions and granzyme release (12). B cells may hinder recovery and cause late-onset cognitive problems after brain damage (13). Natural killer cells can directly kill neurons or interact with other cells to participate in injury (14). In summary, lymphocytes that enter the brain early after ischemic stroke play a key role in making brain damage worse.

In the field of immunology, the P2X7 signaling pathway has been extensively investigated. Studies have shown that P2X7 modulation of energy metabolism is closely associated with T cell activation, cytokine secretion, differentiation, migration, motility, and apoptosis induction (5, 15). P2X7 receptor activation stimulates TCR-mediated calcium influx, NFAT activation, and IL-2 production (16). It also promotes the polarization of CD4 Naïve T cells into Th1/Th17 cells (15). In B cells, P2X7 receptor engagement is typically linked to an active phenotype, migratory behavior, pro-inflammatory effects, and IgM synthesis (17). NKT cells can be activated by P2X7 stimulation; however, they are also susceptible to P2X7-induced cell death (18). Activation of the P2X7 receptor impairs antitumor activity and reduces IFN-γ secretion in natural killer cells (19). The role may differ greatly in distinct immune microenvironments and diseases.

Currently, it is reported that P2X7 activation exacerbates stroke damage via neutrophil extrusion (4). Research on P2X7 regulation of other infiltrating immune cells, particularly lymphocytes, in cerebral infarction is still lacking. The expression levels of P2X7, whether ATP/P2X7 signaling activation in infiltrating lymphocytes and how P2X7 involved in ischemic brain injury have not been systematically investigated. In the present study, we established a transient middle cerebral artery occlusion (tMCAO) mouse model and assessed the expression levels of the P2X7 receptor in infiltrated lymphocytes, particularly in T cells. Our results demonstrate significantly elevated P2X7 expression in CD4^+^T cells and CD8^+^T cells within stroke lesions. Under P2X7 signaling, infiltrated CD4^+^T cells may promote IL-17A secretion, thereby exacerbating ischemic brain injury. These findings provide a potential therapeutic target for the intervention of ischemic stroke.

## 2 Materials and methods

### 2.1 Animals

Wild type (WT) C57BL/6J mice were procured from Hunan SJA Laboratory Animal Co., Ltd. (Hunan, China). P2X7-KO mice, derived from breeding pairs with a C57BL/6J background, were generated by Cyagen Biosciences Inc. (Suzhou, China). Rag2^-/-^ mice were also obtained from Cyagen Biosciences Inc. The animals were housed in a specific pathogen-free (SPF) environment within a light-regulated chamber, adhering to a strict 12-hour light/dark cycle from 8:00 AM to 8:00 PM. The ambient temperature was maintained at 25 ± 2°C with a relative humidity of 50%. All animal experiments conducted in this study were approved by the Animal Ethics Committee of the Army Medical University (formerly known as the Third Military Medical University, Chongqing, China).

### 2.2 Transient focal cerebral ischemia

In this study, a transient middle cerebral artery occlusion (tMCAO) model was employed to induce brain ischemia-reperfusion injury in male mice weighing 20–25 grams. All experimental procedures adhered strictly to the “Regulations for the Protection and Use of Animals” established by the Army Medical University. Inhalation anesthesia was induced by 2% isoflurane (RWD Life Science, China) during surgeries. The detailed protocol is as follows: the mice were secured using medical tape to immobilize their limbs. The fur on the neck region was shaved, and the area was sterilized with an iodine solution. A midline incision approximately 1 cm in length was made along the neck to expose the carotid triangle. Micro forceps were used to carefully dissect the surrounding tissues and nerves attached to the carotid triangle. A rounded-tip nylon suture (Jialing, China) was inserted into the left common carotid artery, advanced into the internal carotid artery until mild resistance was encountered. The mice were maintained on a warming pad for 90 minutes before the suture was withdrawn, thereby completing the procedure. For the sham-operated mice, we performed the same procedures as in the tMCAO group, but without inserting the nylon suture.

### 2.3 Neurological deficit scores

Neurological deficits following reperfusion were assessed using the Longa score test. The scoring criteria are as follows: 0 indicates no neurological deficit; 1 signifies incomplete extension of a paralyzed limb; 2 denotes circling toward the side of paralysis (left); 3 represents falling to the side of paralysis (left); and 4 indicates unconsciousness or an inability to walk. Additionally, the modified neurological severity score (mNSS) was also utilized to evaluate motor function, sensory response, balance, and reflex behavior in the animals. Higher mNSS scores correspond to more severe impairments. Neurological scores range from 0 (normal) to 14 (maximum deficit) (20). Detailed scoring criteria are provided in **Supplementary Table 1**.

### 2.4 Triphenyl-tetrazolium chloride staining

Mice were sacrificed under anesthesia with pentobarbital sodium (50 mg/kg). Then the whole brains were carefully excised and immediately placed in a -80°C freezer for 3 minutes. Subsequently, the brains were sectioned into six slices and promptly transferred to a six-well plate containing 4 mL of a 1% TTC (Sigma, USA) solution. The plate was incubated at 37°C for 20 minutes. Following incubation, the TTC staining solution was discarded, and the slices were fixed overnight in a 4% paraformaldehyde solution. After aspirating the paraformaldehyde solution, the slices were rinsed twice with PBS before being photographed. The percentage of cerebral infarction was quantified using ImageJ software. Specifically, the relative infarct volume (%) was calculated as: total ipsilateral hemispheric ischemic area of each section/total brain area of each section × 100%.

### 2.5 Preparation of brain single-cell suspension

The mice were anesthetized with pentobarbital sodium (50 mg/kg) and perfused with physiological saline via the left ventricle. Ischemic brain hemisphere were excised and collected in 4 mL of DMEM medium. DNase I (Sigma, USA) was added to achieve a final concentration of 0.25 mg/mL, while collagenase D (Roche, Switzerland) was added to reach a final concentration of 0.5 mg/mL. The suspension was incubated in a shaker at 37°C, maintained at 80 rpm for 30 minutes. Subsequently, EDTA buffer (Guangzhou Dingguo Biology, China) was introduced to terminate the enzymatic activity of collagenase D. The digested tissue suspension was filtered through a 40 μm cell strainer and gently triturated until nearly all tissue fragments had passed through. The resulting cell suspension was layered over a 30% Percoll (GE, USA) solution and centrifuged at 2000 rpm for 30 minutes. Cells were washed with PBS and stored at 4°C for subsequent use.

### 2.6 Rotarod test

The rotational speed of the rod was set at 10 revolutions per minute (rpm) for a duration of 2 minutes, repeated twice during for pre-training. The formal experiment consisted of two stages. In the first stage, the speed was maintained at 10 rpm for 30 seconds. In the second stage, the speed was gradually increased from 0 to 40 rpm over a period of approximately 90 seconds, with a maximum testing duration of 300 seconds. The time taken for the mice to fall off the rotating rod during the second stage was recorded. Each trial was separated by an interval of 20 minutes. The average performance across four trials was calculated for each mouse.

### 2.7 Foot fault test

The foot fault test was conducted to evaluate sensorimotor coordination during spontaneous locomotion. Mice were placed on a grid surface (dimensions: 30 cm (L) × 35 cm (W) × 30 cm (H)) with square openings measuring 1.5 cm × 1.5 cm and videotaped for 2 minutes from below the grid. Foot faults were recorded when the mouse misplaced its right limb, causing it to pass through the grid. Each limb movement was counted as one step, and each instance where the right paw missed the grid and fell through was recorded as an error. The foot fault rate was calculated as the percentage of missteps made by the right paw relative to the total number of steps taken by all four limbs (21).

### 2.8 Flow cytometry

A single-cell suspension of brain or spleen was prepared. Subsequently, cell viability staining, surface marker staining, and intracellular factor staining were performed in sequence. For viability staining, the Zombie Yellow™ Fixable Viability Kit (Biolegend, USA) was utilized prior to surface marker staining. Surface marker staining was conducted by incubating the cells at 4°C for 15 minutes. After viability and surface marker staining, the intracellular cytokine detection was conducted with the Cyto-Fast™ Fix/Perm Buffer Set (Biolegend, USA) according to the instruction manual. Cells subjected to intracellular cytokine staining were pre-stimulated with PMA and ionomycin for 2.5 hours. In cases where only surface staining was required, cells were resuspended in Cell Staining Buffer (Biolegend, USA) after PBS washing and proceeded directly to flow cytometric analysis (Beckman, USA). Kalusa 2.1 software (Beckman, USA) was used for data analysis. The threshold for P2X7 was based on P2X7-KO controls, and the gating of other markers were based on cell clusters. All specific antibodies used are listed in **Supplementary Data Sheet 1**.

### 2.9 TUNEL assay

The entire mouse brain was immersed in 5 mL of 4% paraformaldehyde (PFA) for 24 hours. Following fixation, the brain was embedded in paraffin wax and sectioned at a thickness of 5 μm. The sections were then subjected to protease K treatment and permeabilized with 0.1% Triton X-100. Subsequently, a TUNEL reaction mixture containing terminal deoxynucleotidyl transferase (TdT) enzyme and dUTP (AIFang Biological, China) was applied to the sections, followed by incubation at 37°C for 2 hours. Afterward, antigen retrieval was performed, and the sections were incubated with a NeuN antibody (AIFang Biological, China), followed by secondary antibody incubation and DAPI staining. For each sample, three fields of view within the infarct area were selected under 40× magnification to calculate the average value.

### 2.10 Cytokines detection

We collected peripheral serum and prepared tissue homogenates from the infarcted brain hemisphere of the needed mice for cytokine detection. Inflammatory factors of TNF-α, IFN-γ, IL-1β, IL-4, IL-6, GM-CSF, CCL-2, and CCL-4 are detected also by ABplex multi factor detection (ABclonal Technology, China). IL-17A and IL-21 are detected by Mouse ELISA Kit (ABclonal Technology, China) according to the manufacturer’s instructions.

### 2.11 Cell viability assay

The murine hippocampal neuronal cell line HT22, cultured in 96-well plates, was subjected to oxygen and glucose deprivation (OGD) for 6 hours. Following OGD, the HT22 cells were co-cultured with ATP-treated CD4^+^T cells or their conditioned medium supernatant for 24 hours. The CD4^+^T cells were isolated from the spleen of WT and P2X7 knockout mice by magnetic beads. After incubation, the CD4^+^T cells were removed. Subsequently, 10 μL of CCK-8 solution (Baoguang Biotechnology, China) was added to each well, followed by incubation at 37°C for 1.5 hours. The absorbance (A) was measured at 450 nm. Cell viability was calculated using the formula: Viability = (A(experimental) - A(blank))/(A(control) - A(blank)) × 100%.

### 2.12 Transfer of CD4^+^ or CD8^+^ T cells into Rag^−/−^ mice

Male Rag2^−/−^ mice aged 8 to 10 weeks were reconstituted with CD4^+^T cells or CD8^+^T cells. These T cell subsets were isolated from the spleens of WT and P2X7-KO mice using a magnetic separation kit (Miltenyi Biotec, Germany). Before the day preceding tMCAO, 5×10^6^ cells were injected intravenously into the Rag2^-/-^ mice. Subsequently, neurological behavior tests, magnetic resonance imaging (MRI), and behavioral assessments were conducted.

### 2.13 MRI

MRI scans were performed to detect cerebral infarction following tMCAO. On the fourth postoperative day, mice were anesthetized with isoflurane, and MRI imaging was conducted using a 7.0 T BioSpec 70/20USR spectrometer (Bruker, Ettlingen, Germany). This system, equipped with BGA-6S gradients, can achieve a maximum gradient strength of 660 mT/m and a slew rate of 11,250 T/m/s. A total of 22 mouse brain scans were acquired, each with a slice thickness of 0.7 mm. The total scanning duration was 5 minutes and 12 seconds. MRI images were evaluated and analyzed using Radiant Dicom Viewer software. The ischemic volume ratio was calculated as follows: (sum of the white ischemic area in each slice)/(sum of the total area of each brain slice) × 100%.

### 2.14 RNA sequencing

CD4^+^ and CD8^+^ T cells were isolated from the spleens of WT and P2X7-KO mice, and subsequently treated with ATP. RNA extraction was performed using an RNA extraction reagent (ABclonal Technology, China). The extracted RNA was used for library construction, followed by sequencing on the Illumina high-throughput sequencing platform. The image data obtained were converted into raw data using CASAVA base calling software. After quality control and alignment to the reference genome, differential gene expression analysis was conducted using DESeq2, with P-values adjusted according to the Benjamini-Hochberg procedure. Statistical significance was determined based on adjusted P-values and log2 fold changes. Enrichment analysis was performed using the hypergeometric test. For KEGG pathway analysis, pathway-centric hypergeometric distribution assessments were applied, while GO term-based evaluations were conducted for Gene Ontology annotations. Gene set enrichment analysis was performed using the ClusterProfiler R package.

### 2.15 Differentiation of Th17 cells *in vitro*

Prepare the culture medium by combining IMDM medium with L-glutamine, supplemented with 10% fetal bovine serum (FBS) and 1% penicillin/streptomycin solution. The Th17 differentiation conditioned medium was prepared at a 2×concentration, containing 10 ng/mL TGF-β1 (ABclonal Technology, China), 80 ng/mL recombinant IL-6 (ABclonal Technology, China), 20 ng/mL recombinant IL-23 (ABclonal Technology, China), 5 μg/mL anti-IFN-γ (Invitrogen, USA), and 5 μg/mL anti-IL-4 (Invitrogen, USA). Naïve CD4^+^T cells were isolated from the spleens of 8 to 12-week-old mice using the EasySep naïve T cell isolation kit (STEMCELL, Canada). For Th17 cell differentiation, 50 μL of anti-mouse CD3 (2.5 μg/mL) (Biolegend, USA) and anti-mouse CD28 (2.0 μg/mL) (Multi-sciences, China) antibodies were pre-coated in 96-well plates and incubated overnight at 4°C. Prior to cell addition, the plates were washed once with PBS. Each well received 2×10^5^ cells in 100 μL of ordinary culture IMDM medium, and 100 µL 2×conditioned differentiation medium was added as flowing. The cells were cultured in a humidified incubator at 37°C and 5% CO2 for 3 days, and the cells were ready for use (22).

### 2.16 Statistical analyses

Data analysis was conducted utilizing Prism 9.0 software (GraphPad). Statistical significance was assessed via unpaired t-tests, one-way analysis of variance (ANOVA) or two-way ANOVA, depending on the specific experimental design. A p-value threshold of less than 0.05 was established to indicate statistical significance. The levels of significance are represented by asterisks as follows: ns *p*>0.05, *p*<0.05*, *p*<0.01**, *p*<0.001***, *p*<0.0001****.

## 3 Results

### 3.1 P2X7 expression is upregulated in lymphocytes within the infarcted hemisphere

**Figure 1:**
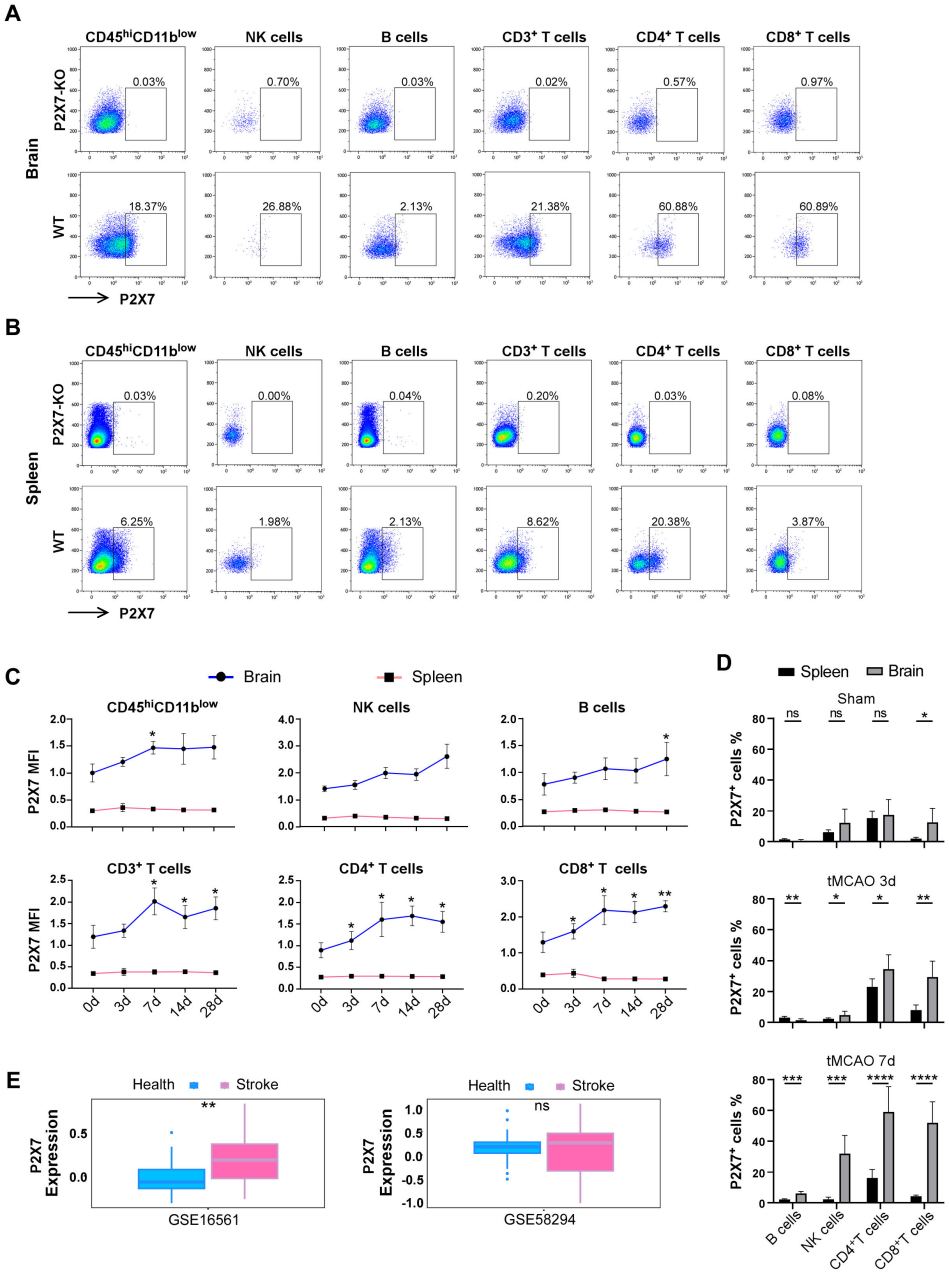
Expression levels of P2X7 receptor in infiltrated lymphocytes of stroke mice. **(A)** Flow cytometry gating of P2X7 on CD45^high^ CD11^blow^ cells, NK cells, B cells, CD3^+^T cells, CD4^+^T cells, and CD8^+^T cells in the brain of WT mice 14 days after tMCAO. P2X7-KO, the threshold of P2X7 which is based on P2X7-KO mice. WT, the gating of P2X7 for the samples. **(B)** Flow cytometry gating of P2X7 on CD45^high^ CD11^blow^ cells, NK cells, B cells, CD3^+^T cells, CD4^+^T cells, and CD8^+^T cells in the spleen of WT mice 14 days after tMCAO. P2X7-KO, the threshold of P2X7 which is based on P2X7-KO mice. WT, the gating of P2X7 for the samples. **(C)** Surface expression levels of P2X7, as measured by mean fluorescence intensity (MFI), on CD45^high^ CD11^blow^ cells, NK cells, B cells, CD3^+^T cells, CD4^+^T cells, and CD8^+^T cells in the spleen and cerebrum at 0, 3, 7, 14, and 28 days post-tMCAO. The asterisks indicate a comparison of the P2X7 MFI values of cells in the brain infarct area with those at 0 d using one-way ANOVA. (n = 5 to 7). The experiment was repeated twice with similar results. **(D)** Percentage of P2X7 on NK cells, B cells, CD4^+^T cells, and CD8^+^T cells in the spleen and cerebrum of sham and tMCAO mice at 3 and 7 days post-surgery (n = 5 to7). Sham, the group of mice that suffer the same procedures of the tMCAO group, but without inserting the nylon suture. The experiment was repeated twice with similar results. **(E)** Analysis of P2X7 expression in peripheral blood samples from healthy individuals and stroke patients using data from Gene Expression Omnibus (GEO) databases (GSE58294, comprising 24 healthy controls and 39 stroke patients. GSE16561, including 23 healthy controls and 69 stroke patients). Data are presented as mean ± SD. Statistical analyses were conducted using multiple unpaired t-tests for panel D and t-tests for panel **(E)** Significance levels: ns, no significant; *p <0.05; **p <0.01; ***p < 0.001, ****p < 0.0001.

To elucidate the expression pattern of P2X7 in lymphocytes during ischemic stroke, we conducted tMCAO on WT C57BL/6J mice (**Supplementary Figures S1A-D**). We subsequently evaluated the expression of P2X7 on lymphocytes from the cerebral infarction hemisphere and spleen at 0, 3, 7, 14, and 28 days post-surgery by flow cytometry (**Figures 1A-D**, **Supplementary Figure S1E**). Analysis revealed that the mean fluorescence intensity (MFI) of P2X7 was significantly higher in NK cells, B cells, and T cells from the cerebral infarction hemisphere compared to those from the spleen in tMCAO mice (**Figure 1C**). Though an upward trend in P2X7 MFI was observed across all lymphocyte populations in the cerebral infarction hemisphere from the acute phase (3 days) through to the chronic phase (28 days), only the MFI of P2X7 was significantly elevated in CD4^+^ and CD8^+^T cells at day 3 and day 7 (subacute stage), as well as throughout the entire duration of brain damage (**Figure 1C**).

Simultaneously, we quantified the percentage of P2X7-positive cells. In the sham group, the percentages of P2X7-positive cells in B cells, NK cells, and CD4^+^T cells exhibited relative lower levels and no significant differences were observed between the brain and spleen (**Figure 1D**). Notably, the proportion of P2X7-positive cells was significantly elevated in both CD4^+^ and CD8^+^T cells during both the acute phase (3 days) and subacute stage (7 days) post-infarction in the brain. This proportion was markedly higher compared to that observed in B cells and NK cells (**Figure 1D**).

Furthermore, we conducted an in-depth analysis of bioinformatics data obtained from the Gene Expression Omnibus (GEO) databases (specifically GSE58294 and GSE16561). Our findings revealed a significant increase in P2X7 expression in the peripheral blood of stroke patients compared to healthy controls (**Figure 1E**). These results corroborate the elevated expression of P2X7 in T cells from cerebral infarction patients and suggest that P2X7 may play a crucial role in ischemic brain pathology.

### 3.2 Deficiency of the P2X7 receptor ameliorates cerebral ischemic injury

**Figure 2:**
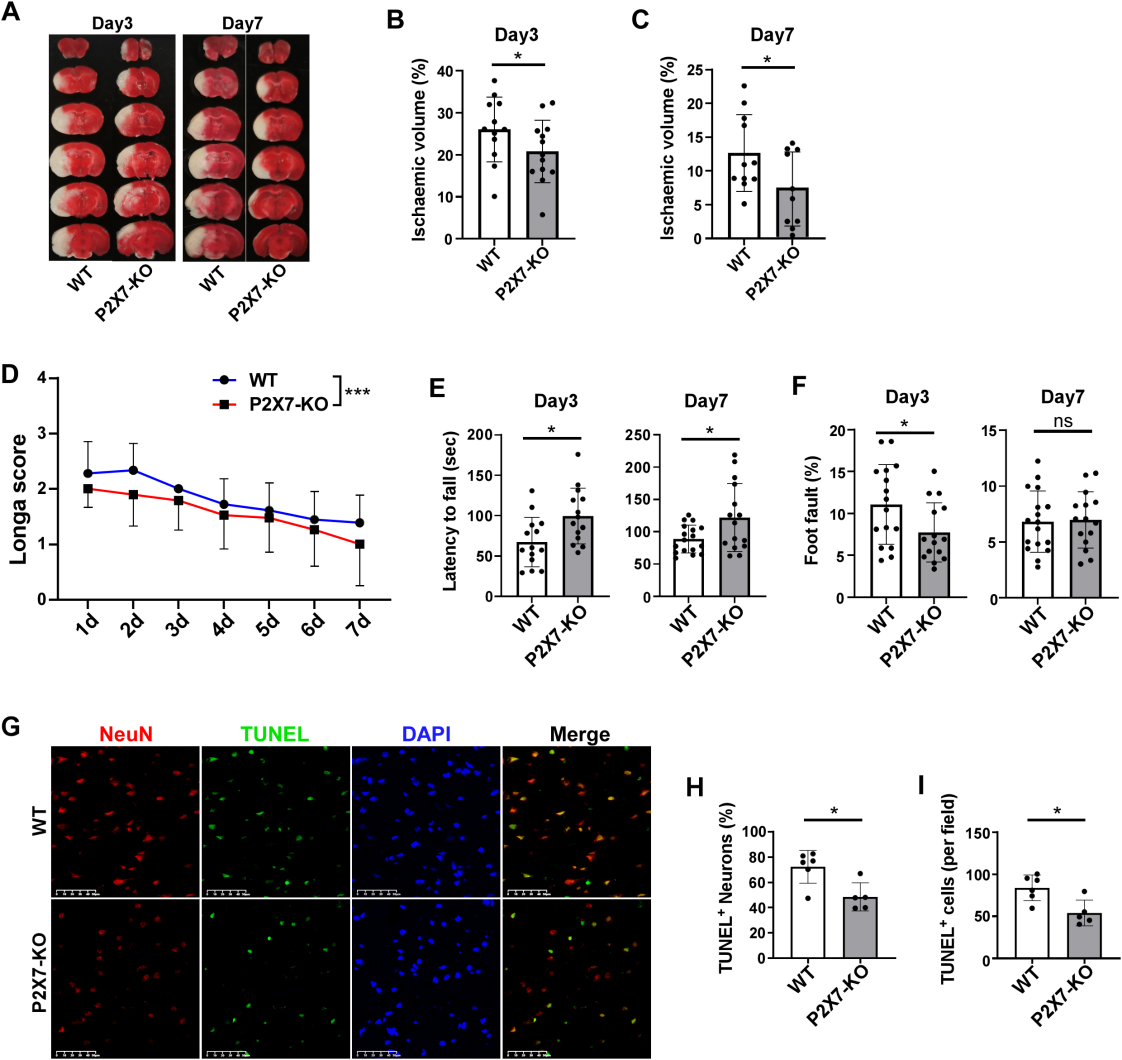
P2X7 receptor deficiency attenuates cerebral ischemic injury. **(A)** Representative TTC-stained coronal brain sections from WT and P2X7-KO mice at 3 and 7 days post-tMCAO. Analysis of relative infarct volumes in WT and P2X7-KO mice at 3 days **(B)** and 7 days **(C)** post-tMCAO. **(D)** Longa score assessed in WT and P2X7-KO mice from 1 to 7 days post-tMCAO. Two-way ANOVA was used to compare the overall changes between the WT and P2X7-KO groups from day 1 to day 7. WT n=18, P2X7-KO n=19. **(E)** Rotarod performance of WT and P2X7-KO mice at 3 and 7 days post-tMCAO. **(F)** Foot fault test results for WT and P2X7-KO mice at 3 and 7 days post-MCAO. **(G)** Representative immunofluorescence images showing TUNEL and NeuN-positive cells in the ischemic penumbra of WT and P2X7-KO mice at 3 days post-tMCAO, Scale bar: 50 μm. WT n=6, P2X7-KO n=5. **(H)** Percentage of TUNEL-positive neurons among all neurons in the ischemic penumbra of WT and P2X7-KO mice at 3 days post-tMCAO, The average value of three fields of view were calculated for each mouse, WT n=6, P2X7-KO n=5. **(I)** Quantification of TUNEL-positive cells per field in the ischemic penumbra of WT and P2X7-KO mice at 3 days post-tMCAO, WT n=6, P2X7-KO n=5. Each point in the bar chart represents the data of an independent mouse sample in the figure. Data are presented as mean ± SD. Statistical analysis was performed using unpaired t-tests **(B, C, E, F, H, I)**. *p <0.05; ***p < 0.001.

Infiltrated lymphocytes play a significant role in promoting post-ischemic inflammation, which contributes to the detrimental effects of acute inflammation in stroke (23). Consequently, our research primarily focuses on the acute and subacute phases following cerebral ischemia. We investigated ischemic damage in WT and P2X7-KO mice after tMCAO. P2X7 expression is low or almost absent in T cells of our P2X7-KO mice (**Supplementary Figure S2**). Our findings revealed that P2X7-KO mice exhibited reduced brain damage compared to WT mice, as evidenced by smaller infarct volumes on both the third and seventh day post-tMCAO (**Figures 2A-D**). Additionally, neurological function assessments using the Longa score and rotarod test indicated better outcomes in P2X7-KO mice to WT mice, although no significant differences were observed in the foot fault test (**Figures 2E, F**). Furthermore, the neuronal apoptosis rate was lower in the P2X7-KO group (**Figures 2G-I**). These results confirm that P2X7 receptor knockout mitigates both acute and subacute cerebral ischemic injury.

### 3.3 P2X7 activation directly exacerbates cerebral damage mediated by CD4^+^T cells following ischemic stroke exposure

**Figure 3:**
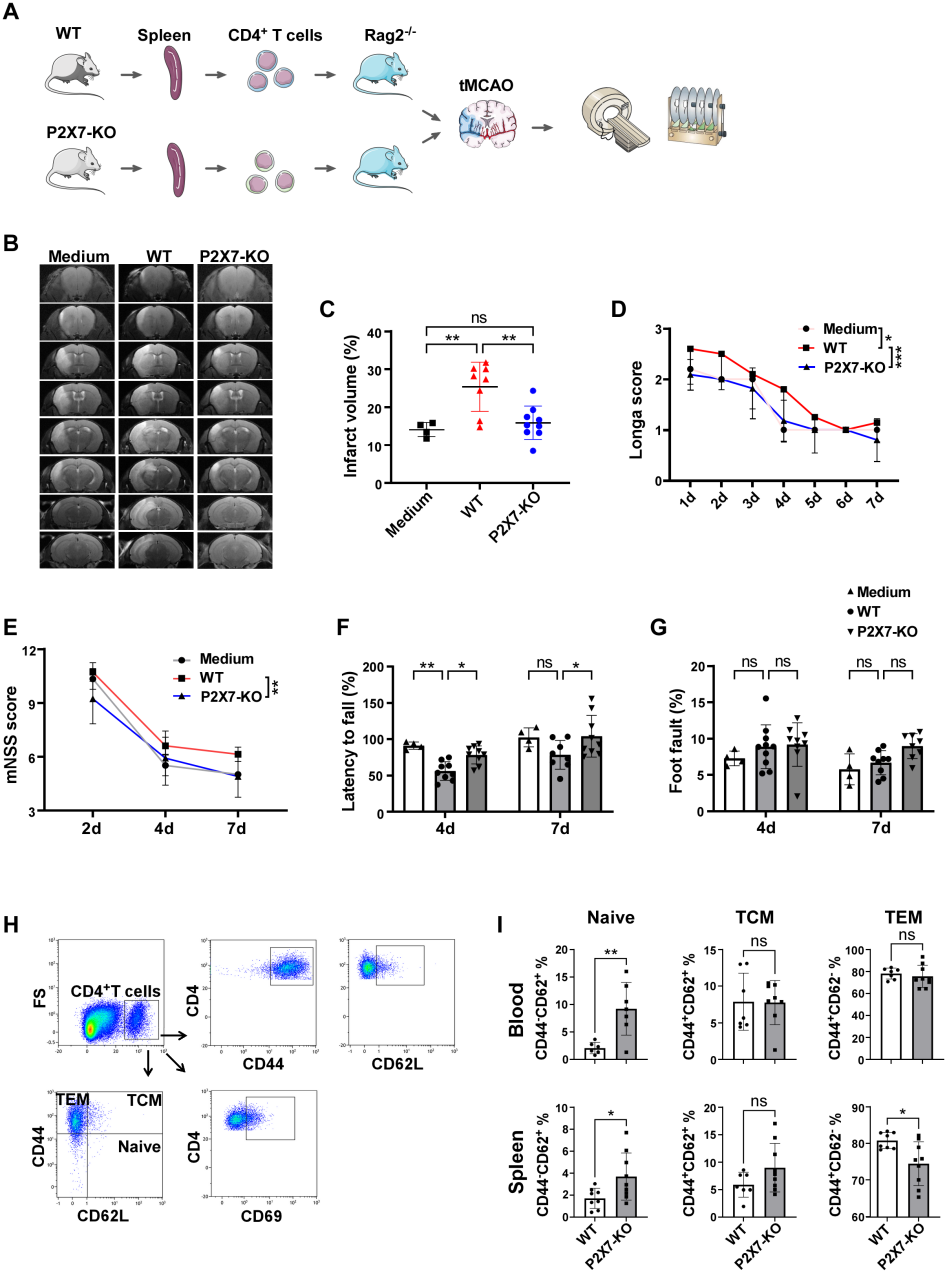
P2X7 directly exacerbates the damage to ischemic cerebral tissues by CD4^+^T cells. **(A)** Schematic diagram of the framework of the transfer experiments design. The splenic CD4^+^T cells were sorted by immunomagnetic beads and were suspended in RPMI 1640 medium. WT CD4^+^T cells, P2X7-KO CD4^+^T cells, or the RPMI 1640 medium were transferred into Rag2^−/−^ mice via the tail vein, followed by tMCAO induction. **(B)** Representative MRI images demonstrating cerebral infarction 4 days post-tMCAO. **(C)** Analysis of relative infarct volumes among the three groups. **(D)** Longa scores for the three groups measured from day 1 to day 7 post-tMCAO. Two-way ANOVA was used to compare the overall changes among the three groups from day 1 to day 7. Medium n=5, WT n=10, P2X7-KO n=11. **(E)** Modified neurological severity scores assessed on days 2, 4, and 7 post-tMCAO. Two-way ANOVA was used to compare the overall changes between the WT and P2X7-KO groups on days 2, 4, and 7 post-tMCAO. Medium n=4, WT n=10, P2X7-KO n=11. **(F)** Rotarod test results on days 4 and 7 post-tMCAO. **(G)** Foot fault test outcomes on days 4 and 7 post-MCAO. **(H)** Flow cytometry gating strategies for CD44, CD62L, CD69, naïve (CD4^+^CD44^-^CD62^+^), TCM (CD4^+^CD44^+^CD62^+^), and TEM (CD4^+^CD44^+^CD62^-^) cells within CD4^+^T cells in recipient mice. **(I)** Frequencies of naïve, TCM, and TEM cells in peripheral blood and splenic CD4^+^T cells. Medium, the control group that injected with only RPMI 1640 medium. WT, the group that injected with WT CD4^+^T cells. P2X7-KO, the group that injected with WT CD4^+^T cells. Each point in the bar chart represents the data of an independent mouse sample in this figure. Data are presented as mean ± SD. Statistical analyses were conducted using one-way ANOVA **(C)**, two-way ANOVA (D to G) and unpaired t-tests **(I)**. Significance levels: ns, no significant; *p <0.05; **p <0.01; ***p < 0.001.

Given the aforementioned findings, we hypothesized that P2X7 may play a direct role in mediating the detrimental effects exerted by CD4^+^ or CD8^+^T cells. To test this hypothesis, CD4^+^ and CD8^+^T cells were isolated from the spleens of WT and P2X7-KO mice and subsequently transferred into Rag2^-/-^ mice, which were then subjected to tMCAO. MRI revealed that the relative cerebral infarct volume was significantly reduced in recipients of P2X7-KO CD4^+^T cells compared to those receiving WT CD4^+^T cells (**Figures 3A-C**). Neurological function was evaluated using the Longa score, modified neurological severity score (mNSS), rotarod test, and foot fault test. The results demonstrated better outcomes in recipients of P2X7-KO CD4^+^ T cells compared to those receiving WT CD4^+^T cells (**Figures 3D-G**). Additionally, we examined tMCAO-induced injury in Rag2^-/-^ mice following adoptive transfer of WT and P2X7-KO CD8^+^T cells. No significant differences in infarct volume or neurological function were observed between these two groups post-surgery (**Supplementary Figure S3**). Collectively, these results indicate that the pathogenic effects of P2X7 are likely mediated through CD4^+^T cells.

Furthermore, the frequency of naïve CD4^+^T cells in the peripheral blood and spleen was significantly elevated in recipients that received P2X7-KO CD4^+^T cells. Splenic CD4^+^ effector memory T cells (TEM) were markedly reduced and no significant differences were observed in central memory T cells (TCM) (**Figures 3H, I**). The expression of the naïve T cell marker CD62L was upregulated in peripheral blood and splenic CD4^+^T cells from the P2X7-KO group, while the activation marker CD44 was significantly downregulated, and no significant difference in CD69 expression between the P2X7-KO and WT groups was observed (**Supplementary Figure S4**).

Overall, Rag2^-/-^ mice that received P2X7-KO CD4^+^T cells exhibited reduced neurological deficits and decreased activation of CD4^+^T cells. These findings confirm that deficiency of P2X7 signaling in CD4^+^T cells directly ameliorates cerebral ischemic damage.

### 3.4 P2X7 participate in mediating the detrimental effects of ischemic stroke though IL-17 and Th17 signaling

**Figure 4:**
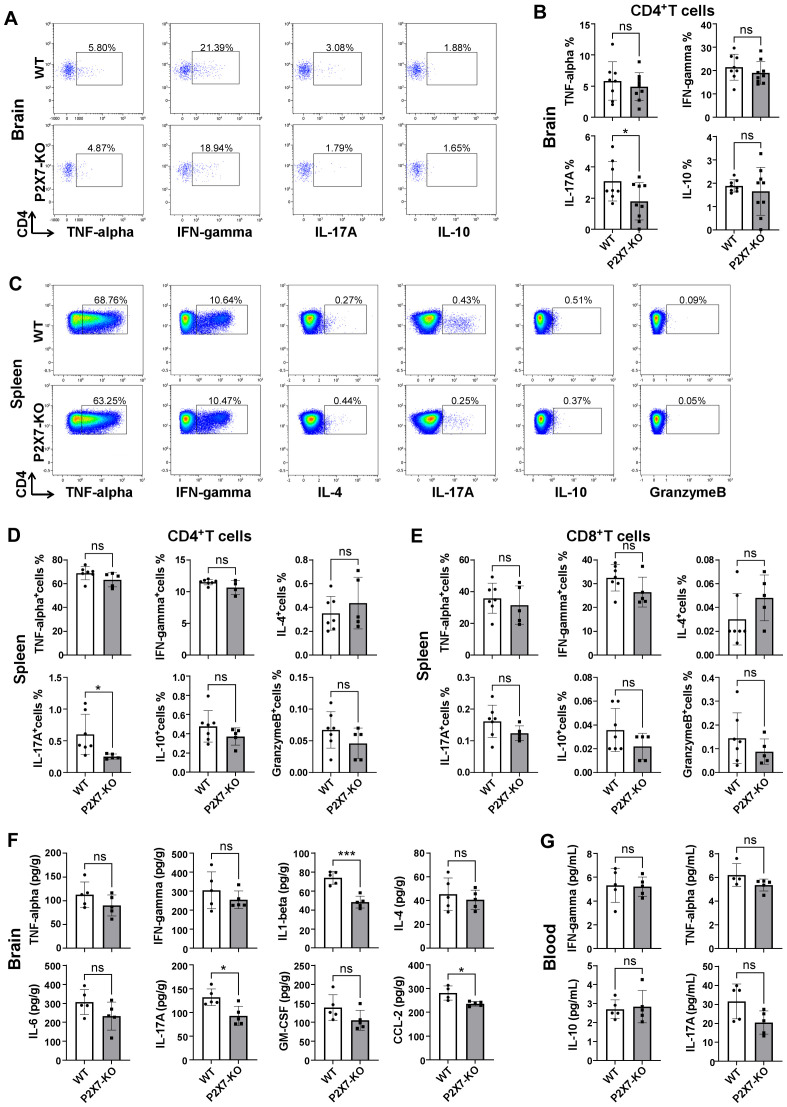
Cytokine in WT and P2X7-KO mice post-MCAO. **(A)** Flow cytometry gating of TNF-α, IFN-γ, IL-17A and IL-10 in cerebral infarction CD4^+^T cells from WT and P2X7-KO mice 7 days post-MCAO. The thresholds of the cytokines were determined by the negative population. **(B)** Frequency of TNF-α, IFN-γ, IL-17A and IL-10 positive cells in cerebral infarction CD4^+^T cells from WT and P2X7-KO mice 7 days post-MCAO. **(C)** Flow cytometry gating of TNF-α, IFN-γ, IL-4, IL-17A, IL-10, and granzyme B expression in splenic CD4^+^T cells from WT and P2X7-KO mice 3 days post-MCAO. The thresholds of the cytokines were determined by the negative population. **(D)** Frequency of TNF-α, IFN-γ, IL-4, IL-17A, IL-10, and granzyme B positive cells among splenic CD4^+^T cells from WT and P2X7-KO mice 3 days post-MCAO. **(E)** Frequency of TNF-α, IFN-γ, IL-4, IL-17A, IL-10, and granzyme B positive cells among splenic CD8^+^ T cells from WT and P2X7-KO mice 3 days post-MCAO. **(F)** Measurement of TNF-α, IFN-γ, IL-1β, IL-4, IL-6, IL-17A, GM-CSF, and CCL-2 in cerebral infarction homogenates from WT and P2X7-KO mice 3 days post-MCAO by ELISA. **(G)** Measurement of IFN-γ, TNF-α, IL-10 and IL-17A in peripheral blood plasma from WT and P2X7-KO mice 3 days post-MCAO by ELISA. Cells subjected to intracellular cytokine staining were resuspended in 1640 medium and pre-stimulated with PMA and ionomycin for 2.5 hours **(A-E)**. Each point in the bar chart represents the data of an independent mouse sample in this figure. Data are presented as mean ± SD. Statistical analyses were performed using unpaired t-tests. ns, no significant; *p <0.05; ***p < 0.001.

Inflammatory cytokines derived from T cells are pivotal in immune regulation. We investigated the levels of various T cell cytokines in the brain and periphery of WT and P2X7 KO mice following ischemic stroke. Flow cytometry analysis showed no significant differences in TNF-α, IFN-γ, and IL-10 levels in cerebral infarction of CD4^+^T cells between WT and P2X7-KO mice 7 days post tMCAO. However, IL-17A was downregulated in the P2X7-KO group (**Figures 4A, B**). And, analysis showed no significant differences in IL-4, IFN-γ, TNF-α, and granzyme B levels in splenic CD4^+^T cells between WT and P2X7-KO mice 3 days post tMCAO. While IL-17A was downregulated in the P2X7-KO group (**Figures 4C, D**). Meanwhile, ELISA results demonstrated that the IL-17A level was significantly decreased in cerebral infarction homogenate and slightly decreased in peripheral blood without significance of the P2X7-KO group (**Figures 4F, G**). There were no significant differences in IL-4, IFN-γ, TNF-α, granzyme B, IL-17A, and IL-10 levels in splenic CD8^+^T cells between WT and P2X7-KO mice (**Figure 4E**). These findings suggest that P2X7 may play a crucial role in regulating IL-17A secretion by CD4^+^T cells and contributes to the exacerbation of cerebral infarction.

**Figure 5:**
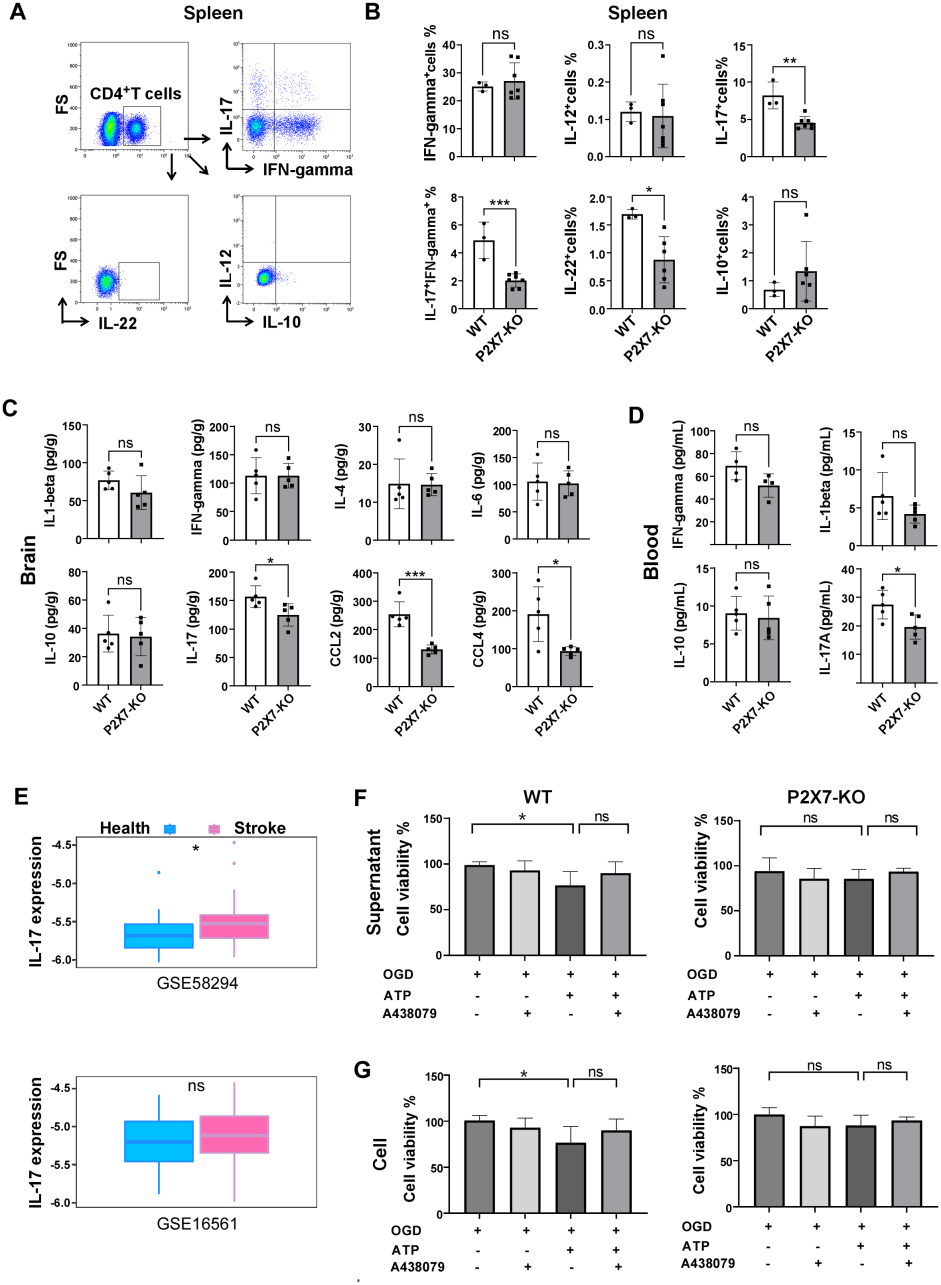
P2X7 may participates in the harmful effects of CD4^+^T cell-mediated ischemic stroke through IL-17A. **(A)** Gating strategies for IL-17A, IFN-γ, IL-12, IL-22, and IL-10 in splenic CD4^+^T cells of the recipient mice by flow cytometry. **(B)** Frequencies of IL-17A, IFN-γ, IL-12, IL-22, and IL-10 positive cells in splenic CD4^+^T cells of Rag2-/- recipient mice 7 days post tMCAO. Splenic cells were resuspended in 1640 medium and pre-stimulated with PMA and ionomycin for 2.5 hours. **(C)** Levels of IL-1β, IFN-γ, IL-4, IL-6, IL-10, IL-17A, CCL-2, and CCL-4 in cerebral infarction homogenates from the Rag2-/- mice receiving the WT or P2X7-KO CD4+T cells measured by ELISA 7 days post-MCAO. **(D)** Concentrations of IFN-γ, IL-1β, IL-10, and IL-17A in peripheral blood plasma from the Rag2-/-mice receiving the WT or P2X7-KO CD4+T cells assessed by ELISA 7 days after MCAO. **(E)** Expression levels of IL-17A in peripheral blood samples from healthy individuals and stroke patients retrieved from the Gene Expression Omnibus (GEO) databases (GSE58294 with 24 healthy controls and 39 patients, and GSE16561 with 23 healthy controls and 69 patients). **(F)** Viability of HT22 cells co-cultured with the supernatant of CD4^+^T cells under different stimulation. n=5 per group. The representative data was from 2 independent experiments. **(G)** Viability of HT22 cells co-cultured with the of CD4^+^T cells under different stimulation. n=5 per group. The representative data was from 2 independent experiments. Data are presented as mean ± SD. Each point in the bar chart represents the data of an independent mouse sample. Statistical analysis was performed using t-tests **(B-D)** and one-way ANOVA **(F)**. ns, no significant; *p <0.05; **p <0.01; ***.

Next, we investigated the cytokines levels in the brain and periphery of Rag2^-/-^ recipient mice. Minimal CD4^+^T cells were detected in control group Rag2^-/-^ mice and the frequency of CD4^+^T cells was similar in the peripheral blood and spleen of WT and P2X7-KO group (**Supplementary Figures S5A-C**). Flow cytometry analysis revealed a significant reduction in the frequency of splenic IL-17^+^CD4^+^T cells, IL-22^+^CD4^+^T cells, and IL-17^+^ IFN-γ^+^CD4^+^T cells in recipients of the P2X7-KO group (**Figures 5A, B**). ELISA results demonstrated decreased levels of IL-17A in both peripheral blood (**Figure 5D**) and cerebral infarction homogenate (**Figure 5C**) of the P2X7-KO group. Furthermore, data from GEO databases (GSE58294 and GSE16561) indicated that IL-17A expression was elevated in the peripheral blood of stroke patients compared to healthy controls (**Figure 5E**). Moreover, the survival rate of OGD-treated HT22 neurons co-cultured with ATP-stimulated WT CD4^+^T cells (**Figure 5G**) or the supernatant (**Figure 5F**) was reduced. However, ATP treatment had no effect on P2X7-KO CD4^+^T cells to impact the viability of HT22 cells.

These findings indicate that the loss of P2X7 signaling may hinder the activation of CD4^+^T cells and inhibit their polarization into Th17 cells, ultimately leading to an amelioration of ischemic brain injury.

### 3.5 P2X7/STAT3/IL-21 signaling pathway plays a critical role in the polarization of Th17 cells

**Figure 6:**
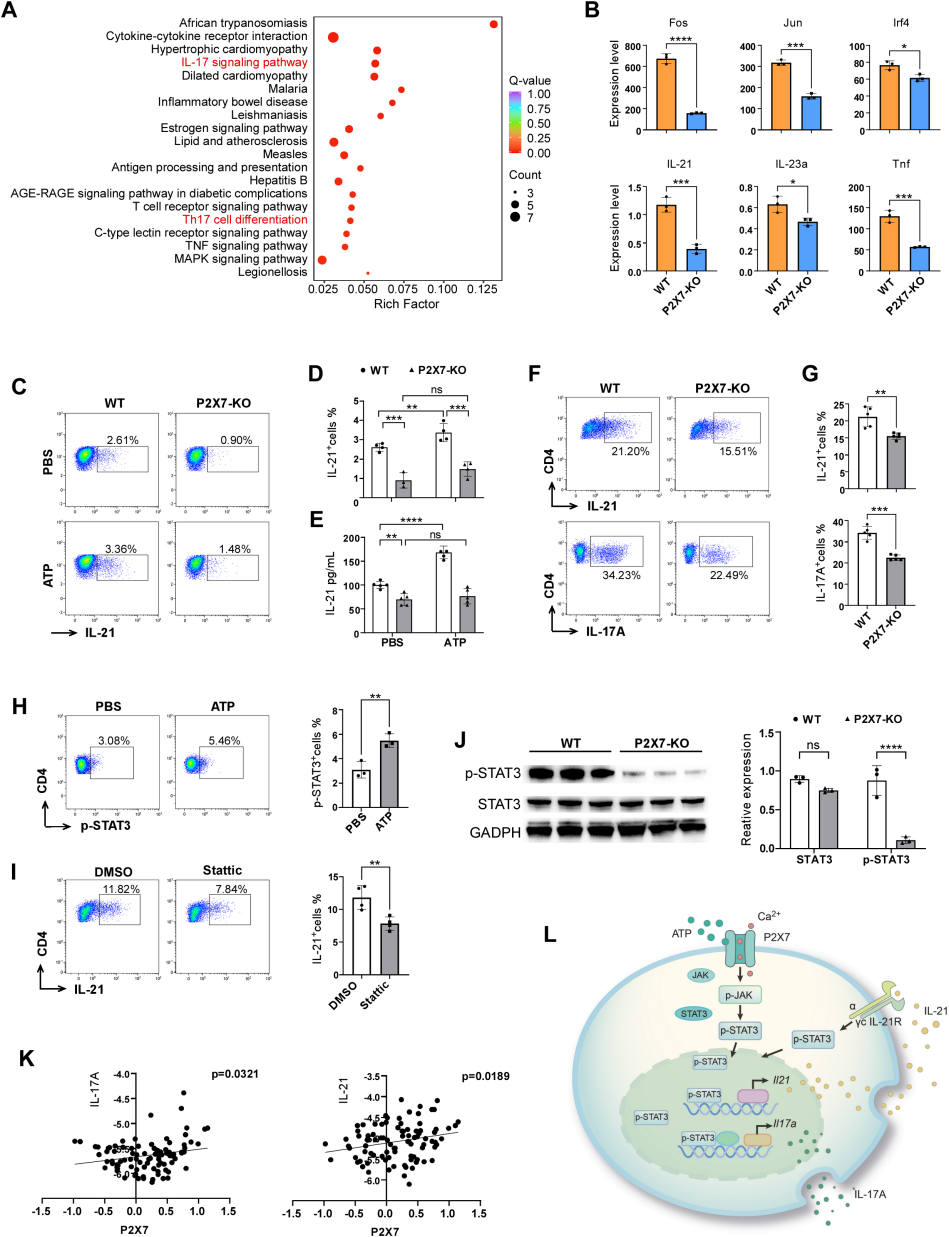
Molecular mechanism of P2X7 signaling-mediated Th17 cell polarization. **(A)** KEGG enrichment analysis of the top 20 pathways comparing P2X7-KO and WT CD4^+^T cells of the transcriptome sequencing. **(B)** Genes down-regulated in the IL-17 pathway in WT and P2X7-KO CD4^+^T cells according to the transcriptome sequencing. **(C, D)** Expression levels of IL-21 in splenic CD4^+^T cells following ATP treatment. Splenocyte were prepared and stimulated with T cell activation cocktail (Biolegend, USA) and 100ng/mL IL-6 (ABclonal Technology, China, China) with or without 300 μM ATP for 6 hours, then IL-21 were assessed by flow cytometry. **(E)** Concentration of IL-21 in the supernatant of splenic sorted CD4^+^T cells after ATP treatment by ELISA test. **(F, G)** Expression levels of IL-21 and IL-17A in WT and P2X7-KO CD4^+^T cells cultured under Th17 differentiation conditions for 3 days. The cells were pre-stimulated with PMA and ionomycin for 2.5 hours before flow cytometry staining. **(H)** Phosphorylation level of STAT3 in CD4^+^T cells following ATP treatment. **(I)** Level of IL-21 in CD4^+^T cells treated with 20 μM STAT3 inhibitor Stattic (MCE, USA). CD4^+^T cells were isolated and activated by anti-CD3/CD28 (Biolegend, USA), 100ng/mL IL-6 for 24 hours with or without Stattic, then T cell activation cocktail were added for another 5 hours. Next IL-21 were assessed by flow cytometry. **(J)** Levels of STAT3, and pSTAT3 in WT and P2X7-KO CD4^+^T cells. WT and P2X7-KO CD4^+^T cells were isolated and stimulated by 20ng/mL IL-6 for 1 hour, then cells were collected and the p-STAT3 was assessed by WB. **(K)** Correlation between P2X7 and IL-17A or P2X7 and IL-21 in PBMCs from stroke patients based on GEO database analysis. **(L)** Schematic representation of P2X7 regulation of IL-17A through the STAT3/IL-21 signaling pathway. The representative data was from 3 independent experiments (**C** to **J**). Each point in the bar chart represents the data of an independent mouse sample. Data are presented as mean ± SD. Statistical significance was determined using t-tests (**B**, **F** to **J**) and two-way ANOVA **(D, E)**. ns, no significant; *p <0.05; **p <0.01; ***p < 0.001, ****p < 0.0001.

Previous studies have indicated that P2X7 signaling in CD4^+^T cells may contribute to the polarization of Th17 cells and participate in ischemic brain injury. To gain a comprehensive understanding of the mechanisms mediated by P2X7, we sorted the splenic CD4^+^ cells from WT and P2X7 KO mice following ATP treatment and conducted the transcriptome sequencing. KEGG enrichment analysis revealed significant enrichment in cytokine receptor interaction, Th17 cell differentiation, and IL-17 signaling pathways when comparing P2X7-KO and WT CD4^+^T cells (**Figure 6A**). Among the nine pathways with the lowest q-values selected for chord diagram analysis, it was evident that the IL-17 signaling pathway was significantly down-regulated in P2X7-KO CD4^+^T cells (**Supplementary Figure S6**). Genes such as *IL-21, Fos, Jun, Irf4, IL-23a*, and *Tnf* were also significantly down-regulated in P2X7-KO cells (**Figure 6B**). It has been reported that IL-21 is predominantly expressed by CD4^+^T cells and functions as an autocrine cytokine essential for Th17 differentiation (24). Based on our sequencing data and previous research, we hypothesize that P2X7 may regulate the secretion of IL-21 in CD4^+^T cells, thereby influencing the synthesis of IL-17A and the differentiation of Th17 cells.

To confirm the impact of P2X7 signaling on IL-21 expression, we examined IL-21 levels in splenic WT and P2X7-KO CD4^+^T cells following ATP treatment. Results of flow cytometry and ELISA revealed that ATP significantly increased IL-21 secretion in WT CD4^+^T cells but not in P2X7-KO CD4^+^T cells (**Figures 6C-E**). To further explore the role of P2X7 in IL-21 expression and Th17 cell differentiation, we induced naive WT and P2X7-KO CD4^+^T cells to Th17 cells. The frequencies of CD4^+^IL-17A^+^ and CD4^+^IL-21^+^cells derived from P2X7-KO T cells were lower than those from WT cells (**Figures 6F, G**). These findings indicate that P2X7 deficiency may impair Th17 polarization by reducing IL-21 expression.

STAT3 is essential for IL-21 production in CD4^+^T cells (25), and it has been reported that P2X7 antagonists inhibit STAT3 phosphorylation in CD4^+^T cells (26). Our findings revealed an elevated level of phospho-STAT3 (p-STAT3) in CD4^+^T cells following ATP stimulation (**Figure 6H**). Upon addition of the STAT3 inhibitor Stattic, the expression of interleukin-21 in CD4^+^T cells was significantly reduced (**Figure 6I**). Additionally, we observed that both STAT3 and p-STAT3 levels were markedly decreased in P2X7-KO CD4^+^T cells compared to WT CD4^+^T cells (**Figure 6J**).

Furthermore, we analyzed the human stroke database GSE58294 and identified a positive correlation between the expression levels of P2X7 and IL-17A, as well as P2X7 and IL-21 (**Figure 6K**). These findings suggest the potential for P2X7 signaling to regulate the expression of IL-21 and IL-17A genes in humans. Therefore, P2X7 may play a crucial role in Th17 cell differentiation through the STAT3/IL-21 signaling pathway (**Figure 6L**).

## 4 Discussion

As an extracellular danger signal, ATP is extensively released during brain ischemia. Following tMCAO, ATP levels in the ischemic hemisphere begin to rise within 30 minutes and remain higher for at least 24 hours compared to the contralateral hemisphere (27). Elevated eATP levels in the ipsilateral striatum persist up to 3 days post-ischemia (28). The overall expression of P2X7 receptors increases in ischemic brain tissue from 6 hours to 7 days post-stroke (29). Previous studies have focused on the effects of P2X7 in resident brain cells under ischemic conditions. However, the role of P2X7 in infiltrating lymphocytes remains unexplored. In this study, we examined the expression patterns of P2X7 in infiltrating lymphocytes and investigated its effects and potential mechanisms in mediating ischemic brain inflammation.

We observed that the expression of P2X7 in splenic lymphocytes is relatively low, yet it is significantly elevated in corresponding cells within stroke lesions. In the infarcted area, P2X7 is highly expressed in CD4^+^ and CD8^+^T cells rather than in B cells or NK cells. P2X7 channel activation results in calcium ion influx and triggers a cascade of downstream responses. P2X7 mediates T lymphocyte activation, cytokine and chemokine release, T cell survival and differentiation, and cell death (5, 30, 31). We next focused on investigating the influence of P2X7 on CD4^+^and CD8^+^T cells in ischemic stroke.

Subsequently, we investigated cerebral infarction damage in P2X7 knockout mice and WT mice. The literature presents varying results regarding the impact of P2X7 deficiency on ischemic stroke. Our findings indicate that P2X7 deficiency mitigates cerebral ischemic injury, which aligns with the results reported by Carlos Matute’s group (32). Another study showed no significant differences in stroke lesion size in WT and P2X7 knockout mice (33). Michael Schäfer’s team noted increased early edema but similar lesion sizes and markedly reduced microglia activation in P2X7 KO mice (34). Two additional studies confirmed that mice overexpressing P2X7 exhibited larger infarcts and poorer neurological outcomes compared to WT groups (35, 36). Furthermore, several investigations have demonstrated that pharmacological blockade of the P2X7 receptor, including the use of various inhibitors, antagonists, and nanobodies, resulted in neuroprotective effects and reduced lesion size (37). Despite inconsistent outcomes from studies using P2X7 knockout mice, the overall consensus is that P2X7 plays a detrimental role in ischemia-reperfusion injury (37).

To further elucidate the direct influence of P2X7 on CD4^+^and CD8^+^T cells, we isolated these cells from WT or P2X7-KO mice and transferred them into Rag2^-/-^ mice followed by tMCAO. No significant differences in infarct volume or neurological function were observed between the two groups with adoptive transfer of CD8^+^T cells. This suggested that P2X7 may not functional through CD8^+^T cells in stroke. However, recipients receiving P2X7-KO CD4^+^T cells exhibited reduced ischemic-reperfusion injury and a decreased frequency of IL-17A^+^CD4^+^T cells and IL-17A^+^IFN-γ^+^CD4^+^T cells compared to recipients of WT cells. IL-17A and Th17 cells play critical roles in stroke pathogenesis. Th17 cells and IL-17A exacerbate blood-brain barrier (BBB) damage, promote infiltration of peripheral immune cells, and increase neuronal injury following ischemic stroke (11, 38). Patients with ischemic stroke exhibit elevated levels of Th17 cells and IL-17A in their peripheral blood, which correlate positively with disease severity, poor prognosis, and post-stroke complications (38). In animal models of ischemic stroke, Th17 cell and IL-17A levels are also increased in both the brain and circulation (11, 39). Some studies have demonstrated that IL-17A secreted by gamma delta T cells serves as a crucial source in stroke pathogenesis (40). Other researches have also observed the upregulation and pro-inflammatory effects of Th17 in the brain (41). But the specific regulatory mechanisms of IL-17 remain unclear. Our findings confirm that P2X7 deletion in CD4^+^T cells reduces the frequency of Th17 cells and mitigates ischemic brain injury. The administration of IL-17A neutralizing antibodies and IL-17A knockout decreased the severity of stroke-induced brain injury (42, 43). And targeting IL-17A transcription, RNA modification or the translation could decrease infarct volume and ischemia reperfusion injury (11, 42). Rendering this a well-established fact in the field, we did not perform the *in vivo* experiments by IL-17A antibody neutralization or using IL-17A knockout mice.

Although those studies have demonstrated that P2X7 enhances IL-17A production in CD4^+^ T cells, the underlying mechanisms remain unclear. Furthermore, we endeavored to elucidate the mechanism by which P2X7 regulates IL-17. Transcriptome sequencing revealed that IL-21 may serve as a critical mediator in the P2X7 signaling pathway involved in IL-17A secretion. IL-21 is an autocrine cytokine essential for the differentiation and maintenance of Th17 cells (24). Our *in vitro* experiments demonstrated that P2X7 activation upregulates IL-21 expression in CD4^+^T cells, while the frequency of IL-21^+^CD4^+^T cells was reduced in P2X7 knockout mice. We have further confirmed that P2X7 plays a positive role in Th17 cell differentiation *in vitro* via the STAT3/IL-21 signaling pathway. Additionally, the regulation of IL-21 by P2X7 may also involve calcium signaling. P2X7 enhances initial Ca^2+^ influx during T cell activation and plays a crucial role in NFAT-1 translocation (30, 44). The 5’-regulatory region of IL-21 contains three NFAT binding sites, and NFAT or NFATc2 directly activate IL-21 transcription (45). Further experiments are required to validate this hypothesis.

Besides, exposure of mature T cells to nicotinamide adenine dinucleotide (NAD+, the substrate for ADP-ribosylation), P2X7 could be ADP-ribosylated and causing calcium flux, pore formation, then finally leading to apoptosis and NAD-induced cell death (NICD) (7, 46). NAD+ released upon cell lysis. This may introduce potential uncertainty in experiments when comparing WT and P2X7-KO T cells. While we employed the C57BL/6 mice, which exhibit much lower sensitivity to ATP-mediated death and NICD comparing BALB/c mice (47). And, we performed cell preparations on ice, which significantly reducing P2X7 mediated cell death (48, 49). Moreover, all the flow cytometry analyses were gated on live cells. Those ensuring a relative accuracy of the results. Therefore, as with the similar studies (50, 51), we did not focus excessively on P2X7 mediated cell death. In the adoptive transfer experiments of the Rag2^-/-^ mice, although the total CD4^+^T cell frequency was comparable between WT and P2X7-KO group in peripheral blood and spleen, we could not completely rule out the effects of P2X7-mediated cell death. NICD-sensitive WT CD4^+^T cell subsets such as Tregs may show a decreased survival rate, which may also be involved in the increase of Th17 response in Rag2^-/-^ recipients. Importantly, this mechanism is still P2X7-dependent and does not alter our main conclusion that the P2X7 receptor plays a significant role in mediating the detrimental effects of ischemic stroke via IL-17 and Th17 signaling pathways.

Additionally, alongside P2X7, other P2X receptors are presented in CD4^+^ T cells, specifically, P2X4 (30). Although the roles of other P2X receptors cannot be entirely ruled out, in our experiments, we employed P2X7 selective inhibitor and P2X7 deficient mice and revealed significant changes that indicate the participation of P2X7 receptor. Meanwhile, although we have demonstrated the significance of IL-17A, we also observed reduced levels of IL-1β and CCL2 in the cerebral infarction homogenates of P2X7-KO mice following tMCAO. IL-1β and CCL2 have a promoting effect on post-stroke inflammation (52, 53). Therefore, the potential contribution of these factors cannot be entirely ruled out and further verification is still needed.

There are several limitations in the present study that warrant acknowledgment. Firstly, *in vivo* experiments involving conditional knockout of P2X7 in CD4^+^T cells have not been conducted. Secondly, the effects of the P2X7 receptor on γδT cells, another source of IL-17A, as well as other relevant cell types during ischemic stroke, remain unexplored. Thirdly, evidence is required to elucidate the precise regulatory mechanisms of P2X7 signaling on IL-21 and IL-17A, and we did not perform antibody neutralization assays or conduct the *in vivo* experiments using knockout mice for IL-17A or IL-21. Further, the influence of P2X7 on infiltrated lymphocytes during chronic stroke has not been observed. In addition, we did not focus on P2X7-mediated T cell death, especially for NICD-sensitive Treg cells, which may have potential impacts. Therefore, future research should address these limitations.

In conclusion, our study highlights that the P2X7 receptor may modulate the secretion of IL-17A in CD4^+^T cells, thereby exacerbating acute and subacute inflammation following stroke. STAT3/IL-21 appears to be an important downstream molecule in P2X7 signaling. Our findings provide novel insights into P2X7-based therapeutic strategies for stroke.

## Data Availability

The raw sequence data in this paper have been deposited in the Genome Sequence Archive in National Genomics Data Center, China National Center for Bioinformation / Beijing Institute of Genomics, Chinese Academy of Sciences (GSA: CRA026860) that are publicly accessible at https://ngdc.cncb.ac.cn/gsa/browse/CRA026860.

## Supplementary material

Supplementary Figure 1 Construction of tMCAO mouse model and the flow cytometry gating of lymphocytes infiltrated in the brain. **(A)** Cerebral blood flow in tMCAO mice under laser speckle contrast imager. **(B)** Representative images of coronal brain sections stained with TTC 3 days post-tMCAO. **(C)** Longa scores of WT mice 3 days after tMCAO. **(D)** Performance on the rotarod test of WT mice at 3 and 7 days post-tMCAO. **(E)** Flow cytometry gating strategies to identify CD45^high^ CD11^blow^ cells, NK cells, B cells, CD3^+^T cells, CD4^+^T cells, and CD8^+^T cells in the brain of WT mice 14 days after tMCAO. Each point in the bar chart represents the data of an independent mouse sample. Data are presented as mean ± SD. Statistical analysis was conducted using unpaired t-tests. ***, p < 0.001.

Supplementary Figure 2 Expression of P2X7 in WT and P2X7 knockout mice. Flow cytometric analysis of P2X7 expression in the spleen of WT and P2X7 KO mice, including gating strategy **(A)** and statistical results **(B)**. n=3 per group. The representative data was from 3 independent experiments. **(C)** Western blot analysis of P2X7 expression levels in the spleen of WT and P2X7-KO mice. n=3 per group. The representative data was from 2 independent experiments.

Supplementary Figure 3 The role of P2X7 signaling in CD8^+^ T cells in cerebral ischemic damage. **(A)** Schematic representation of the experimental design for the transfer experiments. Splenic CD8^+^T cells sorted by immunomagnetic beads were suspended in RPMI 1640 medium. WT CD8^+^T cells, P2X7-KO CD8^+^T cells, or the RPMI 1640 medium were transferred into Rag2^−/−^ mice via the tail vein, followed by tMCAO induction. **(B)** Representative MRI images demonstrating cerebral infarction 4 days post-tMCAO. **(C)** Analysis of relative infarct volumes across the three groups. **(D)** Longa neurological severity scores for the three groups from 1 to 7 days post-tMCAO. Two-way ANOVA was used to compare the overall changes between groups from day 1 to day 7. Medium n=6, WT n=8, P2X7-KO n=8. **(E)** Rotarod performance test results at 4 and 7 days post-tMCAO. Medium, the control group that injected with only RPMI 1640 medium. WT, the group that injected with WT CD8^+^T cells. P2X7-KO, the group that injected with WT CD8^+^T cells. Each point in the bar chart represents the data of an independent mouse sample. Data are presented as mean ± SD. Statistical analysis was performed using one-way ANOVA **(C)** or two-way ANOVA **(D, E)**. ns, no significant; **, p < 0.01.

Supplementary Figure 4 Expression of activation markers in peripheral blood and splenic CD4^+^T cells. Each point in the bar chart represents the data of an independent mouse sample. Data are presented as mean ± SD. Statistical analysis was performed using t-tests. ns, no significant; *, *p <*0.05; **, *p <*0.01.

Supplementary Figure 5 Analysis of CD4^+^ T cells in blood and spleen of Rag2^−/−^ recipient mice. The flow cytometry gating **(A)** and the frequency statistics of CD4^+^T cells in blood **(B)** and spleen **(C)** of Rag2^−/−^ recipient mice. Data are presented as mean ± SD. Statistical analysis was performed using one-way ANOVA. ns, no significant.

Supplementary Figure 6 Chord diagram analysis of the top nine pathways with the lowest q-values in P2X7-KO versus WT CD4^+^ T cells.
