# Supplementary figures and images for: ATP/P2X7 receptor signal aggravates ischemic stroke injury by activating Th17 cells via STAT3/IL-21 pathway
Source: Front Immunol. 2025 Aug 28;16:1558307. doi: 10.3389/fimmu.2025.1558307 (PMC12422926; doi:10.3389/fimmu.2025.1558307)

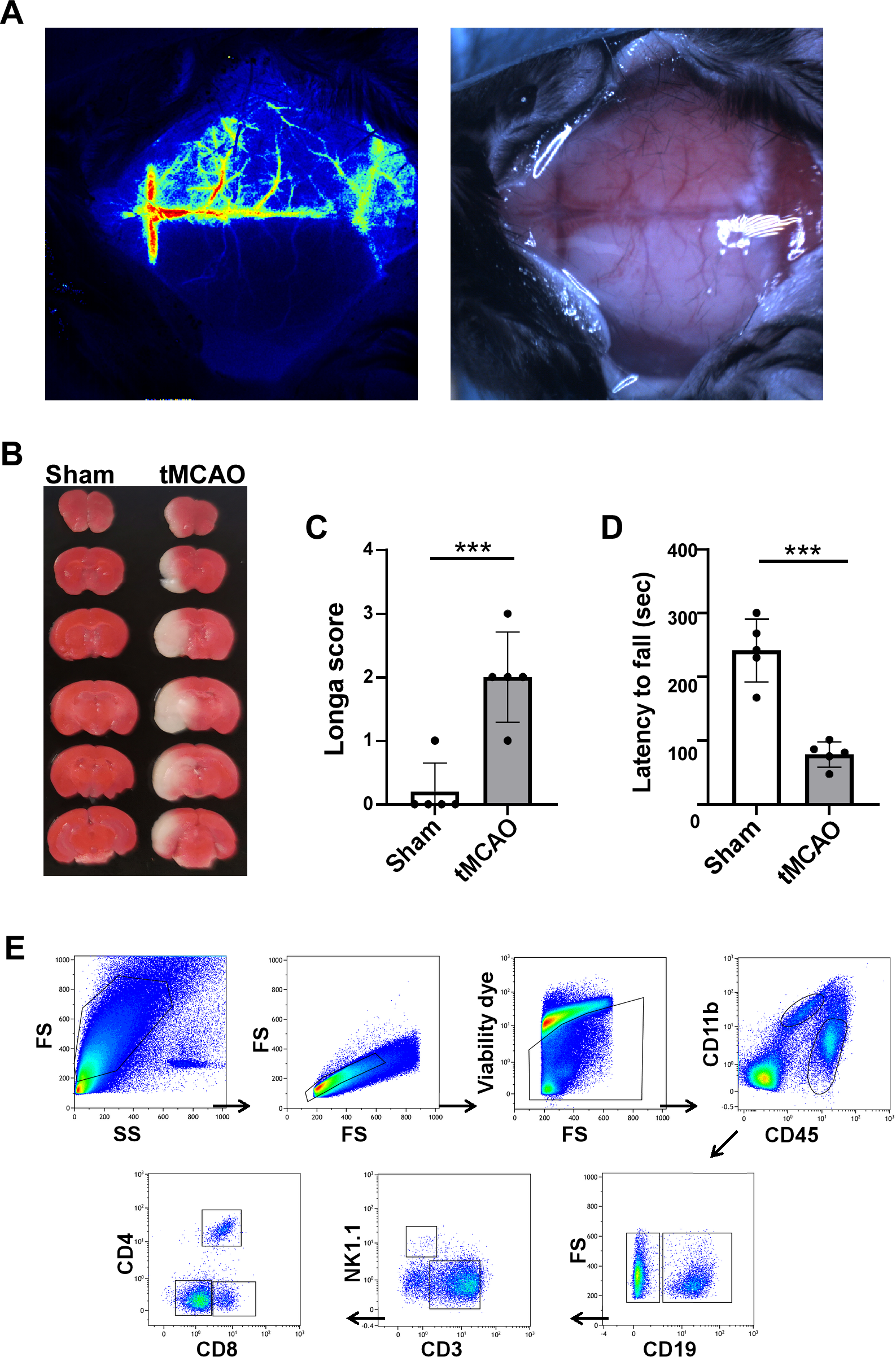

Supplement: Supplementary Figure 1 — Construction of tMCAO mouse model and the flow cytometry gating of lymphocytes infiltrated in the brain. (A) Cerebral blood flow in tMCAO mice under laser speckle contrast imager. (B) Representative images of coronal brain sections stained with TTC 3 days post-tMCAO. (C) Longa scores of WT mice 3 days after tMCAO. (D) Performance on the rotarod test of WT mice at 3 and 7 days post-tMCAO. (E) Flow cytometry gating strategies to identify CD45high CD11blow cells, NK cells, B cells, CD3+T cells, CD4+T cells, and CD8+T cells in the brain of WT mice 14 days after tMCAO. Each point in the bar chart represents the data of an independent mouse sample. Data are presented as mean ± SD. Statistical analysis was conducted using unpaired t-tests. ***, p < 0.001. [file Image1.tif]

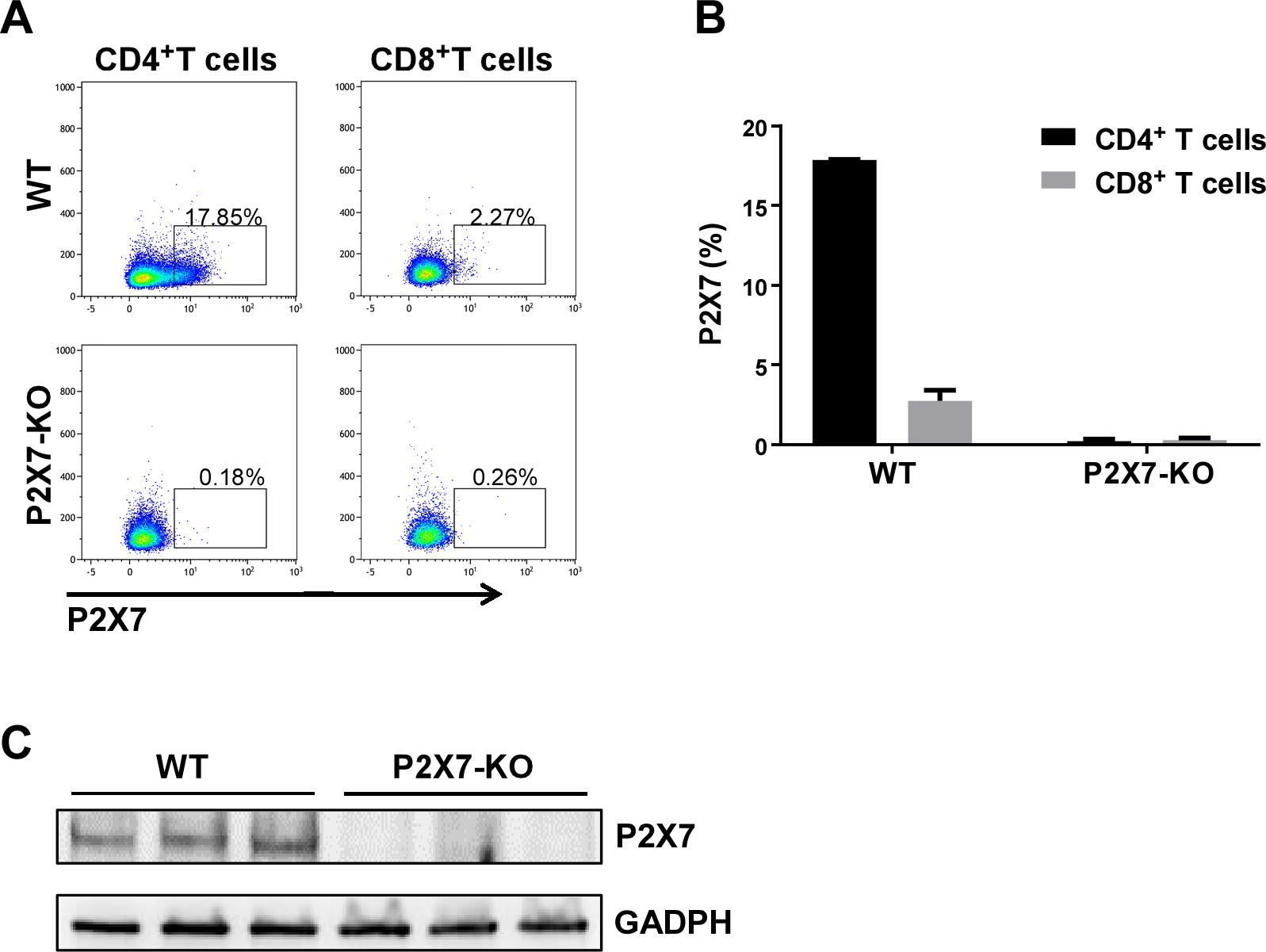

Supplement: Supplementary Figure 2 — Expression of P2X7 in WT and P2X7 knockout mice. Flow cytometric analysis of P2X7 expression in the spleen of WT and P2X7 KO mice, including gating strategy (A) and statistical results (B). n=3 per group. The representative data was from 3 independent experiments. (C) Western blot analysis of P2X7 expression levels in the spleen of WT and P2X7-KO mice. n=3 per group. The representative data was from 2 independent experiments. [file Image2.tif]

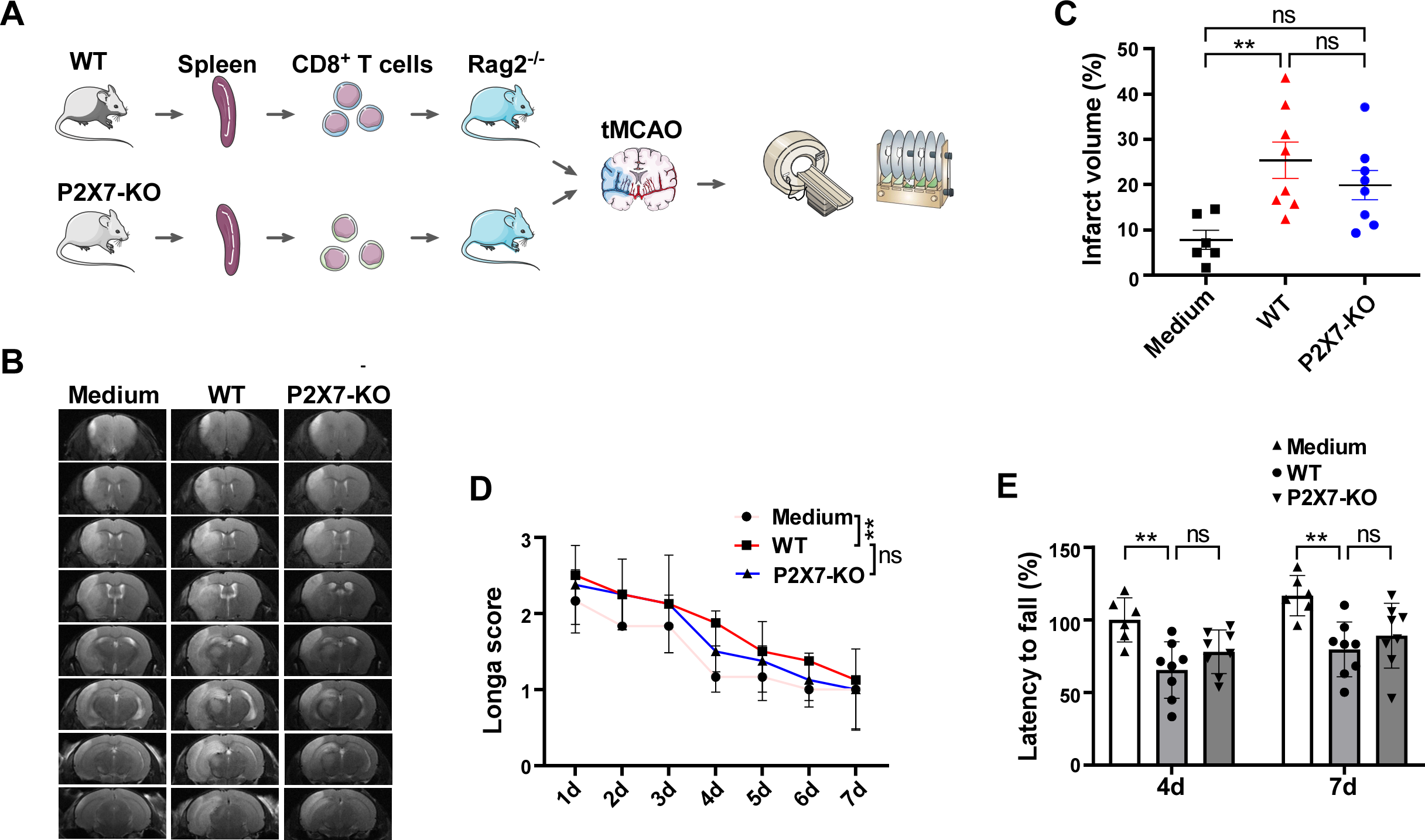

Supplement: Supplementary Figure 3 — The role of P2X7 signaling in CD8+ T cells in cerebral ischemic damage. (A) Schematic representation of the experimental design for the transfer experiments. Splenic CD8+T cells sorted by immunomagnetic beads were suspended in RPMI 1640 medium. WT CD8+T cells, P2X7-KO CD8+T cells, or the RPMI 1640 medium were transferred into Rag2−/− mice via the tail vein, followed by tMCAO induction. (B) Representative MRI images demonstrating cerebral infarction 4 days post-tMCAO. (C) Analysis of relative infarct volumes across the three groups. (D) Longa neurological severity scores for the three groups from 1 to 7 days post-tMCAO. Two-way ANOVA was used to compare the overall changes between groups from day 1 to day 7. Medium n=6, WT n=8, P2X7-KO n=8. (E) Rotarod performance test results at 4 and 7 days post-tMCAO. Medium, the control group that injected with only RPMI 1640 medium. WT, the group that injected with WT CD8+T cells. P2X7-KO, the group that injected with WT CD8+T cells. Each point in the bar chart represents the data of an independent mouse sample. Data are presented as mean ± SD. Statistical analysis was performed using one-way ANOVA (C) or two-way ANOVA (D, E). ns, no significant; **, p < 0.01. [file Image3.tif]

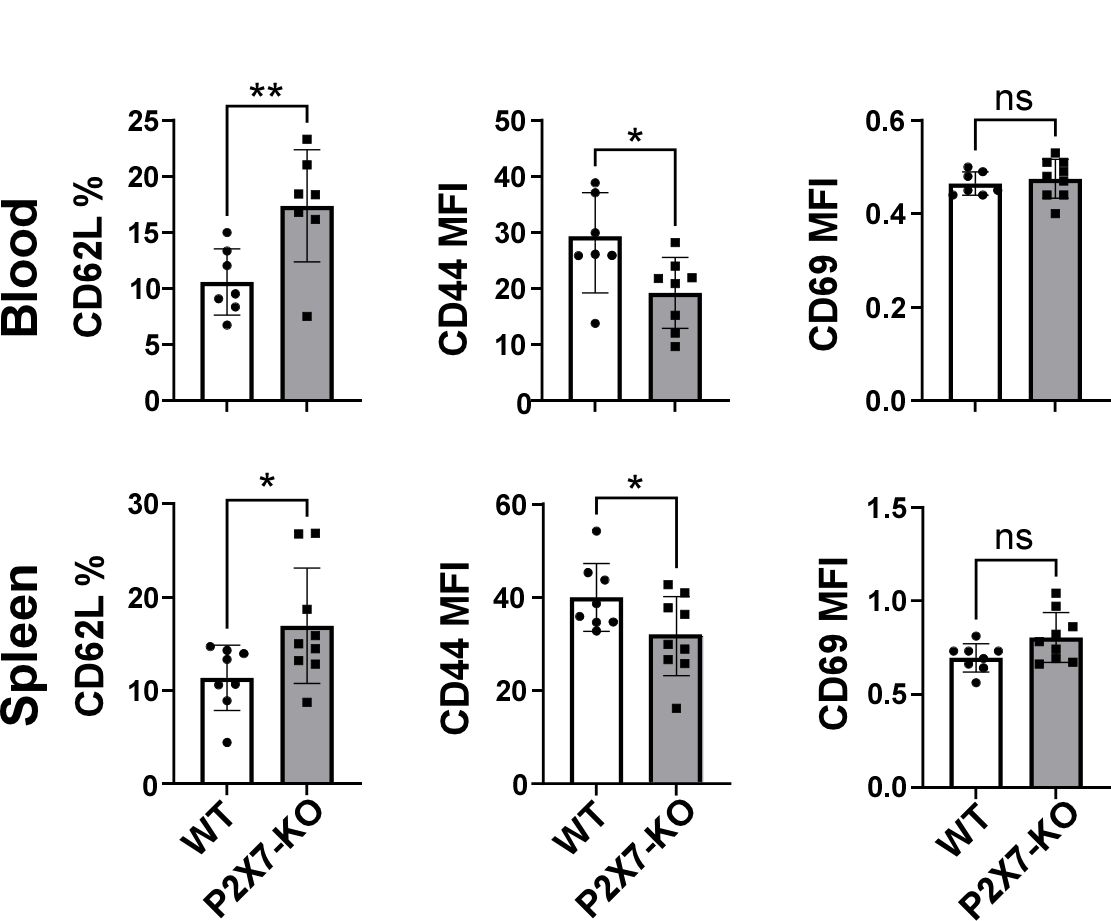

Supplement: Supplementary Figure 4 — Expression of activation markers in peripheral blood and splenic CD4+T cells. Each point in the bar chart represents the data of an independent mouse sample. Data are presented as mean ± SD. Statistical analysis was performed using t-tests. ns, no significant; *, p <0.05; **, p <0.01. [file Image4.tif]

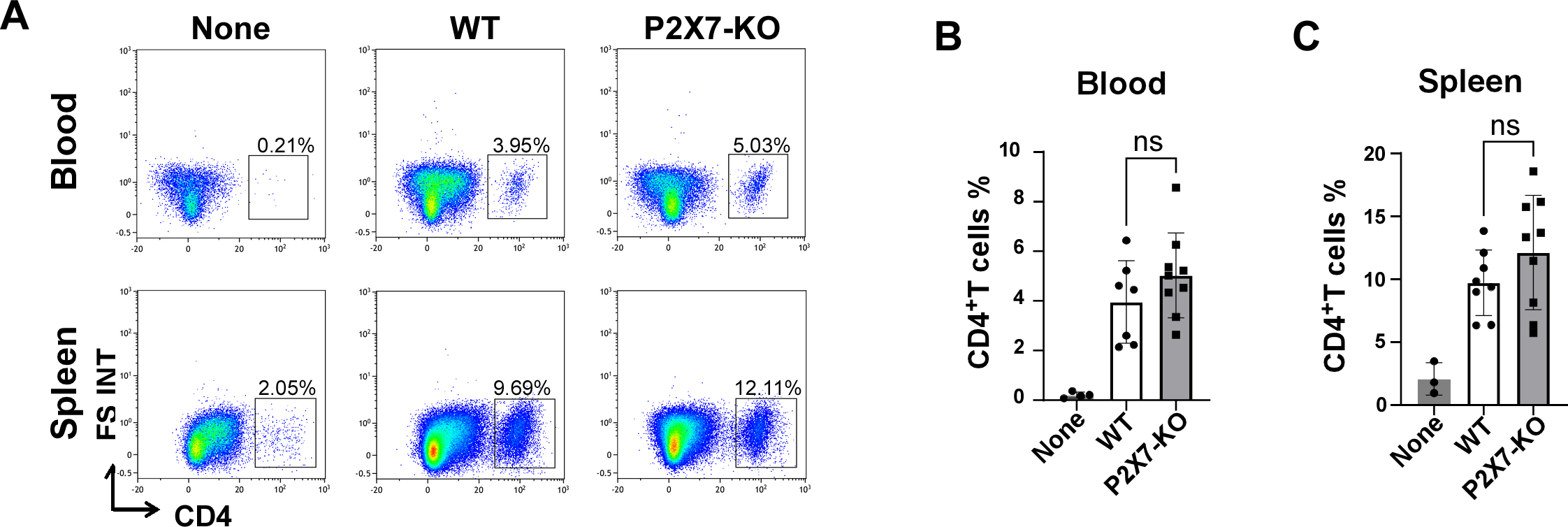

Supplement: Supplementary Figure 5 — Analysis of CD4+ T cells in blood and spleen of Rag2−/− recipient mice. The flow cytometry gating (A) and the frequency statistics of CD4+T cells in blood (B) and spleen (C) of Rag2−/− recipient mice. Data are presented as mean ± SD. Statistical analysis was performed using one-way ANOVA. ns, no significant. [file Image5.tif]

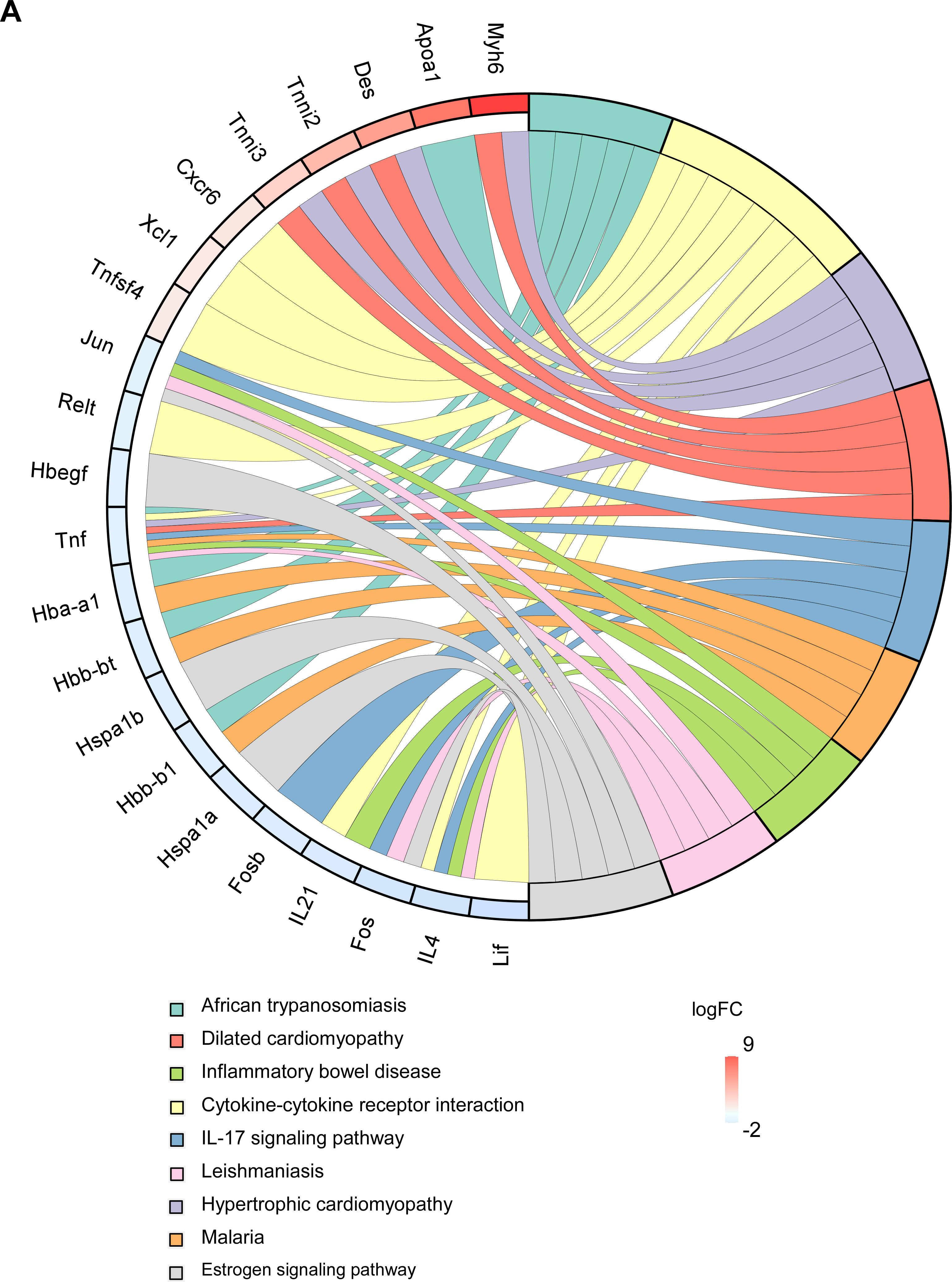

Supplement: Supplementary Figure 6 — Chord diagram analysis of the top nine pathways with the lowest q-values in P2X7-KO versus WT CD4+ T cells. [file Image6.tif]
