# Supplementary material for: ATP/P2X7 receptor signal aggravates ischemic stroke injury by activating Th17 cells via STAT3/IL-21 pathway
Source: Front Immunol. 2025 Aug 28;16:1558307. doi: 10.3389/fimmu.2025.1558307 (PMC12422926; doi:10.3389/fimmu.2025.1558307)
Supplement: Supplementary file 8 [file Table1.docx]

**Supplementary Table 1. The modified neurological severity scoring criteria.**

| **Projects** | **Criteria** | **Scoring** |
| --- | --- | --- |
| **Tail lifting** | Forelimb flexion | 1 |
|  | Limb flexion | 1 |
|  | Head rotation deviates from the central axis by more than 10 ° within 30 seconds | 1 |
| **Place the mouse on the ground** | Walk normally | 0 |
|  | Cannot go straight | 1 |
|  | Rotate towards the paralyzed side | 2 |
|  | Tilt towards the paralyzed side | 3 |
| **Balance beam test** | Maintain a stable state of balance | 0 |
|  | Grasp the edge of the balance beam tightly | 1 |
|  | Grasp the balance beam tightly but one foot drop from it | 2 |
|  | Grasp the balance beam tightly but drop both feet off the balance beam, or rotate the balance beam (>60s) | 3 |
|  | Attempting to maintain balance on the balance beam, but falling (>40s) | 4 |
|  | Attempting to maintain balance on the balance beam, but falling (>20s) | 5 |
|  | Falling: No attempt to maintain balance or grab the balance beam (<20s) | 6 |
| **Reflex deficiency and abnormal activity** | Ear reflex (shaking head when stimulating the external auditory canal) | 1 |
|  | Corneal reflex (blinking when cotton lightly touches the cornea) | 1 |
| **Total score (14)** | |  |
